# Targeted Chromosomal Insertion of Large DNA into the Human Genome by a Fiber-Modified High-Capacity Adenovirus-Based Vector System

**DOI:** 10.1371/journal.pone.0003084

**Published:** 2008-08-29

**Authors:** Manuel A. F. V. Gonçalves, Maarten Holkers, Gijsbert P. van Nierop, Roeland Wieringa, Maria G. Pau, Antoine A. F. de Vries

**Affiliations:** 1 Virus and Stem Cell Biology Laboratory, Department of Molecular Cell Biology, Leiden University Medical Center, Leiden, The Netherlands; 2 Crucell Holland BV, Leiden, The Netherlands; National Institute on Aging, United States of America

## Abstract

A prominent goal in gene therapy research concerns the development of gene transfer vehicles that can integrate exogenous DNA at specific chromosomal loci to prevent insertional oncogenesis and provide for long-term transgene expression. Adenovirus (Ad) vectors arguably represent the most efficient delivery systems of episomal DNA into eukaryotic cell nuclei. The most advanced recombinant Ads lack all adenoviral genes. This renders these so-called high-capacity (hc) Ad vectors less cytotoxic/immunogenic than those only deleted in early regions and creates space for the insertion of large/multiple transgenes. The versatility of hcAd vectors is been increased by capsid modifications to alter their tropism and by the incorporation into their genomes of sequences promoting chromosomal insertion of exogenous DNA. Adeno-associated virus (AAV) can insert its genome into a specific human locus designated *AAVS1*. *Trans*- and *cis*-acting elements needed for this reaction are the AAV Rep78/68 proteins and Rep78/68-binding sequences, respectively. Here, we describe the generation, characterization and testing of fiber-modified dual hcAd/AAV hybrid vectors (dHVs) containing both these elements. Due to the inhibitory effects of Rep78/68 on Ad-dependent DNA replication, we deployed a recombinase-inducible gene switch to repress Rep68 synthesis during vector rescue and propagation. Flow cytometric analyses revealed that *rep68*-positive dHVs can be produced similarly well as *rep68*-negative control vectors. Western blot experiments and immunofluorescence microscopy analyses demonstrated transfer of recombinase-dependent *rep68* genes into target cells. Studies in HeLa cells and in the dystrophin-deficient myoblasts from a Duchenne muscular dystrophy (DMD) patient showed that induction of Rep68 synthesis in cells transduced with fiber-modified and *rep68*-positive dHVs leads to increased stable transduction levels and *AAVS1*-targeted integration of vector DNA. These results warrant further investigation especially considering the paucity of vector systems allowing permanent phenotypic correction of patient-own cell types with large DNA (e.g. recombinant full-length *DMD* genes).

## Introduction

Experiments in animal models of various human inherited diseases have shown that high-capacity (hc) adenovirus (Ad) vectors are superior to early region-deleted Ad vectors both in terms of safety and persistence of transgene expression [1]–[3]. However, at present, extensive use of this gene delivery system in *in vivo* settings is hampered by the difficulty in obtaining the vast amounts of vector particles needed for large animal experiments and the limited understanding of the physiological effects caused by the interaction(s) between vector capsids and, for instance, the innate immune system. In addition, because Ad genomes normally remain episomal, hcAd vectors are of limited use in applications aiming at stable genetic modification of dividing cell populations. Thus, a high demand exists to endow hcAd vectors with mechanisms that allow exogenous DNA persistence.

A strategy to achieve this goal consists of incorporating into hcAd vector genomes the DNA integration-promoting elements from naturally occurring integration systems such as (i) DNA transposons/transposases, (ii) retroviral long terminal repeats/integrases, (iii) retrotransposons, (iv) DNA recombinases or (v) the Rep78/68-binding sequences/large Rep proteins of the seemingly non-pathogenic helper-dependent parvovirus adeno-associated virus (AAV) [4], [5]. Of these integrating hcAd-based vector systems, those that capitalize on the DNA integration machinery of AAV have the advantage of allowing preferential insertion of foreign DNA into the so-called *AAVS1* locus on human chromosome 19 (19q13.3-qter). AAV *trans*- and *cis*-acting elements needed for this reaction are the Rep78 and/or Rep68 protein and their cognate binding sequences, respectively [6]. The *cis*-acting elements are located in the AAV inverted terminal repeats (ITRs), which can fold into a palindromic T-shaped hairpin, and in the AAV p5 promoter, which contains a sequence dubbed the p5 integration efficiency element (p5IEE) [7].

Another shortcoming of hcAd-based gene transfer systems relates to the fact that many clinically important cell types (e.g., human hematopoietic stem and progenitor cells, mesenchymal stem cells and myoblasts) are refractory to transduction by conventional hcAd vectors, which are based on the species C human Ad (hAd) serotypes 2 and 5. This is explained by the virtual absence of the Ad and Coxsackie B virus receptor (CAR) on the surface of these cells [8]–[11]. To allow efficient transduction of CAR-negative cell types, others and we have deployed a genetic retargeting strategy whereby cell receptor-interacting domains of the hAd serotype 5 (hAd5) fiber are substituted by those from other Ads resulting in hcAd particles pseudotyped with chimeric fibers [11]–[13].

In the present study, we describe the generation of dual hcAd/AAV hybrid vectors (dHVs) [14] with modified capsids and with all the AAV *cis*- and *trans*-acting elements needed for locus-specific insertion of exogenous DNA. To avoid the well-described interference of the large AAV Rep proteins (i.e., Rep78 and Rep68) on the Ad-dependent DNA replication [15], [16], which is required for vector genome amplification, we devised a FLP recombinase-dependent gene switch module to repress and activate *rep68* expression in producer and target cells, respectively. Rep68 synthesis in target cells was induced with the aid of a fiber-modified hcAd vector encoding a thermostable version of the *Saccharomyces cerevisae* FLP recombinase (FLPe) [17], [18]. Long-term cultures and clonal analyses of human cervix carcinoma (HeLa) cells and of myoblasts isolated from a patient with Duchenne muscular dystrophy (DMD) were used to determine stable transduction levels as well as the targeted chromosomal integration frequencies of the large DNA molecules delivered by capsid-modified and *rep68*-containing dHV particles. Activation of the inducible *rep68* gene contained in the dHV genome resulted in increased stable transduction frequencies and was required for the efficient insertion of vector sequences into the *AAVS1* locus. Moreover, Southern blot analyses of stably transduced HeLa cell and DMD myoblast clones suggest that locus-specific vector DNA integration occurs relatively more often in diploid than in genetically unstable target cells. Finally, our results suggest that linear double-stranded DNA molecules may serve as substrates for the locus-specific AAV integration machinery.

## Results

### Experimental strategy

Since AAV Rep68 contains all the structural information needed to catalyze targeted DNA insertion, we decided to focus on the delivery into target cells of this instead of the larger Rep78 protein. However, because Ad-dependent DNA replication is strongly inhibited by the two large Rep proteins [15], [16] *rep68* expression during dHV production should be minimized. Indeed, generation of Ad-based vectors encoding Rep78 and/or Rep68 is not a trivial matter since even relatively low concentrations of these proteins in producer cells lead to the emergence and selection of mutant vectors with deletions in the *rep* sequences [19], and our unpublished results. Another point of concern is the stability of Ad vector genomes containing integration modules consisting of classic recombinant AAV (rAAV) genomes (i.e. ITR-transgene[s]-ITR). Investigations have shown that the presence of pairs of non-terminal inverted repeats in Ad vector genomes leads to replication-induced recombination through these sequences causing deletions/rearrangements in the vector DNA [20], [21]. Ad vectors containing both rAAV genomes and *rep78* and/or *rep68* genes may, in addition, suffer from Rep78/68-mediated rescue (i.e. excision) of the rAAV DNA from a fraction of the vector backbones causing transgene loss. The origin of this problem lies in the fact that, although Rep synthesis can, to a great extent, be inhibited by using stringent inducible gene expression systems, most if not all of them are “leaky” (especially in the context of replicating DNA templates). During the production of *rep78/68*-positive Ad vectors, producer cells will thus contain not only Ad-derived AAV helper activities (i.e. Ad gene products necessary for AAV DNA rescue and replication [6] but also Rep78/68 proteins. Finally, permanent expression of the *rep78/68* gene at high levels is detrimental for many cell types causing cell cycle arrest and apoptosis [22]–[24].

**Figure 1:**
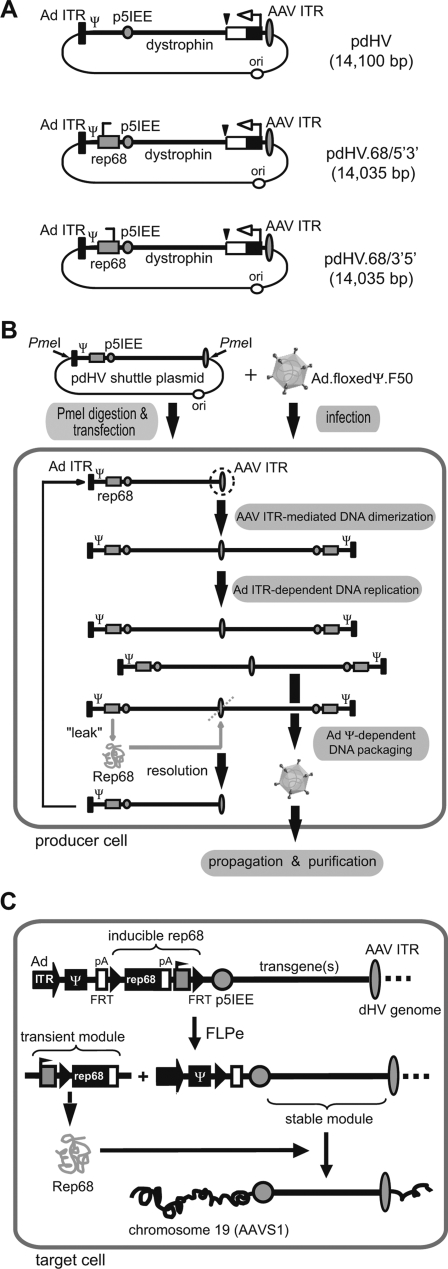
Overview of the structure, production and application of dHVs. (A) Diagram of dHV shuttle plasmids. The structure of pdHV differs from that of pdHV.68/5′3′ and pdHV.68/3′5′ in that the former construct only codes for eGFP whereas the latter plasmids have, in addition, a FLP-inducible AAV *rep68* expression module (see below) inserted in between the Ad packaging signal and the AAV p5IEE in the 5′→3′ (pdHV.68/5′3′) or in the 3′→5′ orientation (pdHV.68/3′5′). Solid box, Ad ITR (hAd5 base pairs 1 through 103); Ψ, Ad packaging signal-containing sequence (hAd5 genome positions 104 through 454); shaded box with broken line, inducible AAV2 *rep68* expression unit; shaded circle, p5IEE (AAV2 genome positions 153 through 291); line with subscript “dystrophin”, stuffer DNA derived from human *dystrophin* cDNA; solid triangle, human *growth hormone* gene pA signal; open box, *eGFP* ORF; solid box with broken arrow, *hEF1α* promoter; shaded oval, AAV ITR (AAV2 nucleotides 1 through 145); open circle, prokaryotic origin of replication. (B) Schematic representation of the rescue process of capsid-modified and *rep68*-positive dHV particles. Rescue of dHV DNA into hAd5 capsids containing chimeric fibers is initiated by releasing it from the plasmid backbone at the indicated PmeI restriction sites and transfecting the digestion products into the hAd5 E1-complementing PER.tTA.Cre76 cells. These cells are next infected with the E1-deleted helper Ad vector Ad.floxedΨ.F50, which supplies *in trans* all Ad DNA replication activities and Ad structural proteins (including chimeric fiber proteins) needed for the amplification and packaging of dHV DNA, respectively. Replication- and packaging-competent tail-to-tail dimers are assembled from input monomers due to Ad-dependent DNA replication and AAV ITR-mediated dimerization. The resulting dHV particles display on their surface hAd50 fiber domains and can be amplified by serial propagation in Ad.floxedΨ.F50-infected producer cells prior to purification in CsCl density gradients. The use of a helper Ad vector which has its packaging signal flanked by bacteriophage P1 *loxP* sites in combination with producer cells expressing the bacteriophage P1 *cre* gene strongly inhibits the formation of helper Ad particles. (C) Transduction pathway of *rep68*-positive dHVs. The FLP-inducible *rep68* expression unit consists of a direct repeat of two FRT sequences flanking the AAV2 *rep68* ORF followed by the herpes simplex virus *thymidine kinase* gene pA signal and the *hGAPDH* promoter. Since the transcription-promoting elements of the *hGAPDH* gene are positioned downstream of the Rep68-coding sequence, the *rep68* ORF is not preceded by an active promoter and hence should not get expressed. To suppress *rep68* activation by possible cryptic promoter elements located upstream of the *rep68* initiation codon, a transcriptional terminator from the murine *metallothionine* gene was introduced immediately upstream of the 5′ FRT site. The FLP-inducible *rep68* expression module is located between the Ad *cis*-acting elements (i.e., the Ad ITR and the Ad packaging signal [Ψ]) and the AAV p5IEE. Excision and circularization of the FRT-flanked DNA by FLPe will position the *hGAPDH* promoter in front of the *rep68* ORF and lead to the synthesis of Rep68. Finally, Rep68-mediated interactions between the RBEs in the vector DNA (i.e., AAV p5IEE and AAV ITR) and in human chromosome 19 (i.e. *AAVS1* locus) will induce targeted chromosomal insertion of dHV DNA.

Given all these potential pitfalls, we decided to construct hcAd-based vectors containing *rep68* genes and Rep78/68-binding elements (RBEs) using a unique vector design in which FLP recombinase-dependent *rep68* expression units are incorporated into the non-integrating parts of dHV genomes [14]. Previous results have shown that Ad-dependent DNA replication in concert with AAV ITR-mediated dimerization is the main mechanism responsible for the generation of dHV genomes from input monomeric templates molecularly cloned in shuttle plasmids [14]. These monomeric DNA molecules contain an Ad ITR, Ad packaging signal and AAV p5IEE at one end and an AAV ITR at the other end (Fig. 1A). The particular design of the resulting dHV genomes with AAV ITR sequences at the axis of symmetry and with the p5IEE at both extremities allows the introduction into target cells of DNA molecules structurally resembling wild-type AAV proviruses [25]. Resolution of the Holliday-like AAV ITR hairpin structures at the axis of symmetry either by cellular factors [14], [26] or by Rep68-mediated nicking at the terminal resolution site (trs; Fig. 1B) will generate monomer-sized replicative intermediates that can be reutilized by the Ad replication machinery to produce new dHV genomes. Because they possess a favorable size for efficient packaging into Ad capsids (i.e. between 27 and 38 kb) [14], [27] unresolved dimeric replicative intermediates will be preferentially packaged into Ad capsids [14] thus giving rise to dHV particles (Fig. 1B). The localization of the FLP-inducible *rep68* gene upstream of the p5IEE in the *rep68*-positive dHV genomes (Fig. 1C) should impede its Rep68-dependent insertion into the *AAVS1* locus [14]. To further reduce the likelihood of integrating *rep68* sequences into the *AAVS1* locus of target cells, the *rep68* expression module was designed in such a way that its FLPe-mediated activation is accompanied by its physical separation from the gene(s)-of-interest. Circularization of the FLP recombinase target (FRT)-flanked DNA by FLPe positions a promoter in front of the *rep68* open reading frame (ORF) resulting in Rep68 synthesis. Finally, Rep68-mediated interactions between the RBEs in the dHV genomes (i.e., AAV p5IEE and AAV ITR) and in human chromosome 19 (i.e. *AAVS1* locus) should facilitate targeted insertion of the foreign DNA (Fig. 1C).

### Construction and testing of a FLP-dependent rep68 expression unit

**Figure 2:**
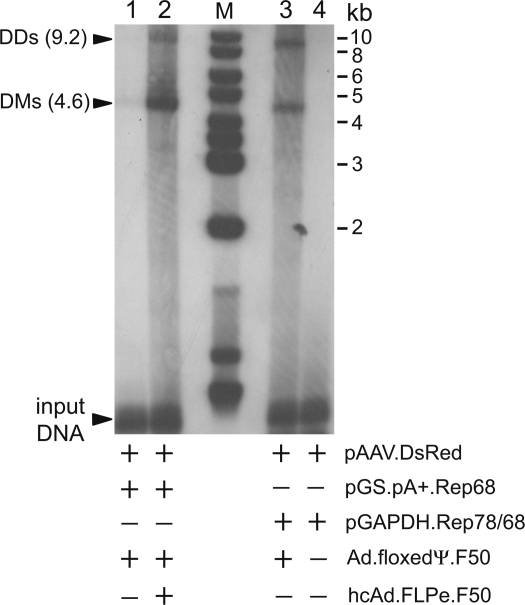
Testing the functionality of pGS.pA+.Rep68 and hcAd.FLPe.F50. Extrachromosomal DNA was extracted from PER.tTA.Cre76 cells co-transfected with the rAAV vector shuttle plasmid pAAV.DsRed and the FLP-activatable *rep68* expression plasmid pGS.pA+.Rep68 and infected 24 h later with Ad.floxedΨ.F50 alone (lane 1) or with Ad.floxedΨ.F50 plus hcAd.FLPe.F50 (lane 2). Positive (lane 3) and negative (lane 4) controls consisted of extrachromosomal DNA extracted from Ad.floxedΨ.F50- and mock-infected PER.tTA.Cre76 cells, respectively, co-transfected with pAAV.DsRed and the constitutive *rep78/68* expression plasmid pGAPDH.Rep78/68. The extracts were treated with DpnI to selectively digest input, non-replicated, prokaryotic DNA and were subjected to Southern blot analysis using a probe corresponding to the *DsRed.T4* ORF. Lane M, GeneRuler DNA Ladder Mix molecular weight marker (Fermentas). The positions and sizes (in kb) of the rAAV replicative intermediates (i.e., DMs and DDs) are indicated. The numerals at the left correspond to restriction DNA fragment sizes in kb.

We started by constructing an inducible *rep68* expression unit on the basis of the FLP/FRT site-specific recombination system of *Saccharomyces cerevisiae*. To this end, the coding sequence for DsRed.T4 in pGS.pA+.DsRed [28] was replaced by that of Rep68 giving rise to construct pGS.pA+.Rep68. The functionality of pGS.pA+.Rep68 was tested in a transient transfection assay. This assay consisted of introducing into pGS.pA+.Rep68-containing PER.tTA.Cre76 cells, AAV helper functions and an rAAV vector shuttle plasmid through infection with Ad.floxedΨ.F50 and transfection of pAAV.DsRed, respectively. In the presence of AAV helper functions plus functional AAV Rep68 molecules, rAAV genomes are expected to be rescued (i.e. excised) from plasmid backbones and to originate *de novo* synthesized replicative intermediates consisting of double-stranded full-length monomeric and dimeric vector DNA, also known as duplex monomers (DMs) and duplex dimers (DDs), respectively [6]. To activate the *rep68*-specific gene switch, we used hcAd.FLPe.F50. Negative and positive controls consisted of pGAPDH.Rep78/68- and pAAV.DsRed-transfected PER.tTA.Cre76 cells that were mock-infected and infected with Ad.floxedΨ.F50, respectively. Results depicted in Fig. 2 clearly show that accumulation of rAAV replicative intermediates in the pGS.pA+.Rep68-transfected PER.tTA.Cre76 cells is strongly stimulated by infection with hcAd.FLPe.F50 (i.e. in the presence of FLPe; compare lanes 1 and 2 of Fig. 2). In the absence of the recombinase only low amounts of rAAV replicative intermediates were generated (Fig. 2; lane 1). This suggests the existence of “leaky” *rep68* expression at a level sufficient to allow limited amplification of rAAV DNA. Indeed, previous research has provided evidence that only few Rep78/68 molecules are required to induce AAV DNA replication [29]. The detection of rAAV-specific DMs and DDs in cells that received pAAV.DsRed, pGAPDH.Rep78/68 and Ad.floxedΨ.F50 (Fig. 2; lane 3) but not in cells containing exclusively pAAV.DsRed and pGAPDH.Rep78/68 (Fig. 2; lane 4) validated the assay. On the basis of these results, we conclude that pGS.pA+.Rep68 is FLPe-responsive and gives rise to the synthesis of functional Rep68 molecules.

### Rescue, propagation and characterization of rep68-positive and fiber-modified dHV particles

**Figure 3:**
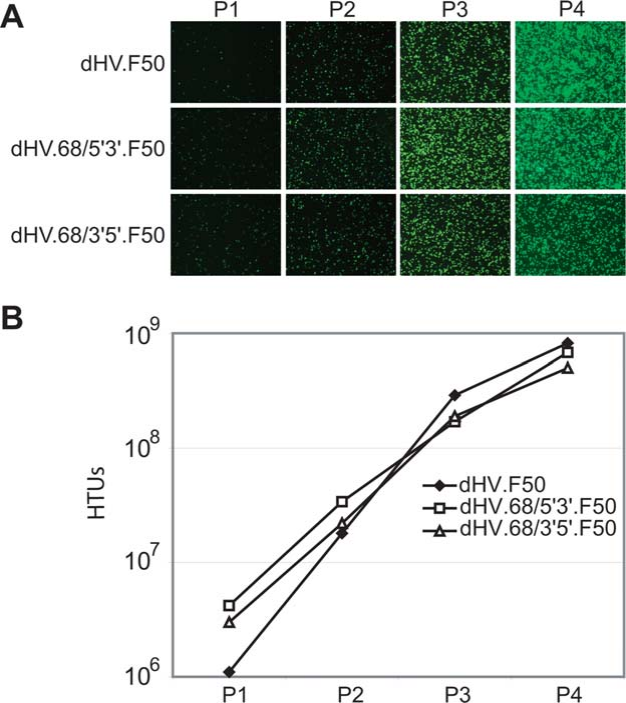
Propagation of *rep68*-positive and fiber-modified dHVs. (A) Direct fluorescence microscopy analysis of the PER.tTA.Cre76 cells used for the serial propagation of dHV.F50, dHV.68/5′3′.F50 and dHV.68/3′5′.F50. (B) Flow cytometric analysis of HeLa cells employed for the determination of the *eGFP* transfer activity of clarified producer cell lysates derived from consecutive rounds of vector amplification. P1, passage 1; P2, passage 2; P3, passage 3; P4, passage 4.

Next, the FLP-inducible *rep68* expression unit from pGS.pA+.Rep68 was inserted in either orientation upstream of the p5IEE in the dHV shuttle construct pAd/AAV.EGFP [14], hereafter referred to as pdHV, to generate pdHV.68/5′3′ and pdHV.68/3′5′, respectively (Fig. 1A). pdHV.68/5′3′, pdHV.68/3′5′ and the *rep68*-negative control plasmid pdHV were digested with PmeI and individually transfected into PER.tTA.Cre76 cells. The PmeI digestion releases the Ad ITRs from almost the complete plasmid backbones turning them into much more efficient substrates for the initiation of Ad-dependent DNA replication than undigested templates. Subsequently, helper activities necessary for the amplification and packaging of dHV genomes were provided in *trans* by the infection of producer cells with the capsid-modified helper Ad vector Ad.floxedΨ.F50. After exhibiting complete cytopathic effect, the cell cultures were harvested and dHV seed stocks were prepared. These crude vector preparations were used to amplify dHV particles through four successive rounds of infection in Ad.floxedΨ.F50-infected PER.tTA.Cre76 cells. Direct fluorescence microscopy revealed an increase in the frequency of enhanced green fluorescent protein (eGFP)-positive producer cells following each consecutive round of vector amplification (Fig. 3A). Quantification of functional vector particles by eGFP-directed flow cytometry showed that the propagation kinetics of the two different *rep68*-positive dHVs was very similar to that of their *rep68*-negative counterpart (Fig. 3B).

**Figure 4:**
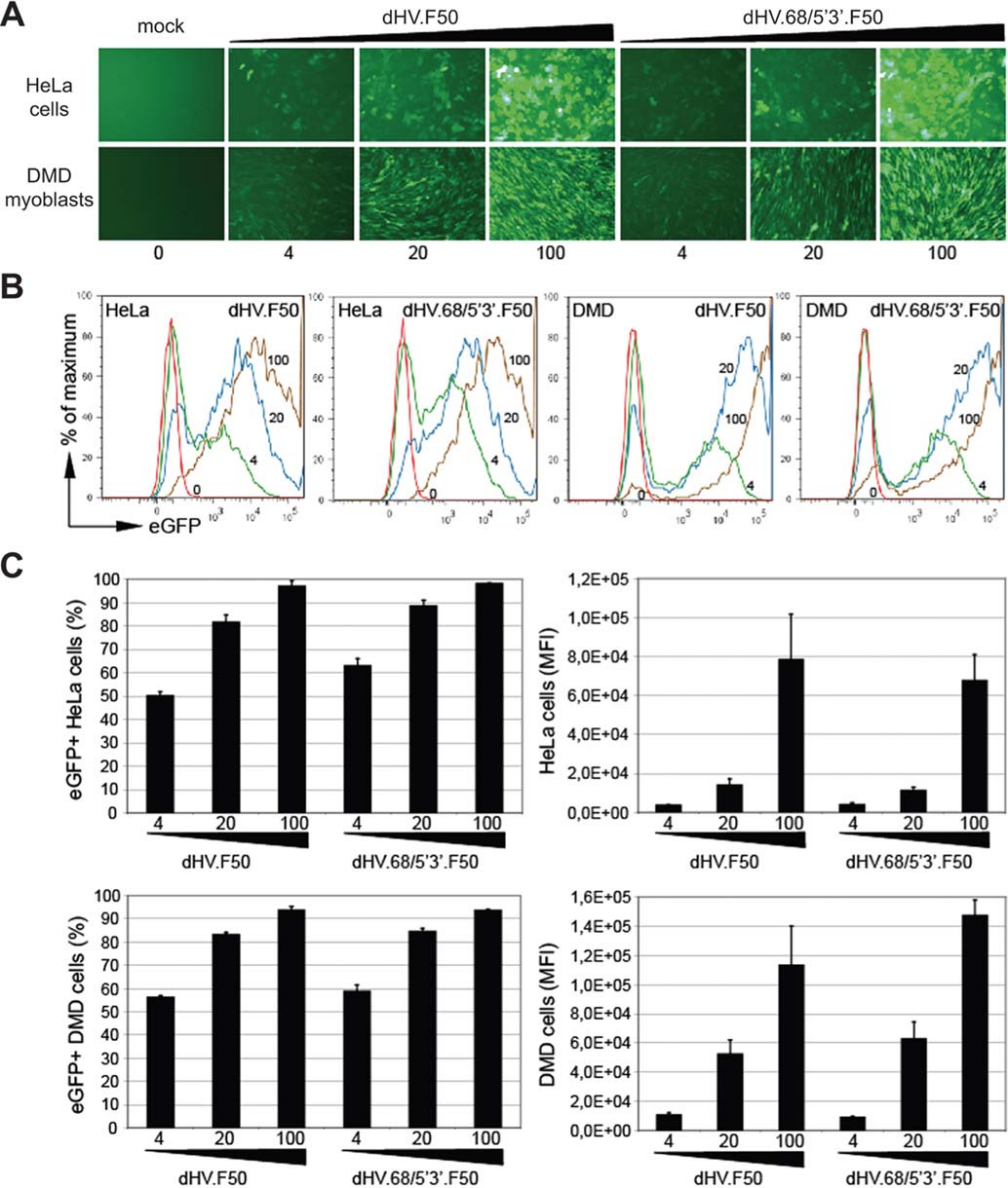
Transduction of target cells by CsCl density gradient-purified dHV.F50 and dHV.68/5′3′.F50 particles. (A) Direct fluorescence microscopy analysis of HeLa cells and DMD myoblasts that were either mock-infected or infected with 4, 20 or 100 HTUs of dHV.F50 or dHV.68/5′3′.F50 per cell. (B) Representative flow cytometry histograms corresponding to HeLa cells and DMD myoblasts transduced with different amounts of dHV.F50 or dHV.68/5′3′.F50 (i.e. 0, 4, 20 or 100 HTUs/cell). The % of maximum refers to the ratio between the number of events analyzed in each case and the number of events in the sample with the largest number of events. (C) Quantification by flow cytometry of gene transfer into HeLa cells and DMD myoblasts exposed to 4, 20 or 100 HTUs of dHV.F50 or dHV.68/5′3′.F50 per cell (*n* = 3). Gene transfer activity is expressed both in terms of the frequency of eGFP-positive cells and the mean fluorescent intensity (MFI) of the transduced cells (which reflects mean intracellular eGFP levels). Error bars represent standard deviations. All measurements were performed at 72 hours postinfection.

Both *rep68*-negative and *rep68*-positive fiber-modified dHV particles obtained after four rounds of propagation were purified by isopycnic CsCl density gradient ultracentrifugation, titrated and used to transduce CD46-positive target cells that either do (HeLa cells) or do not (DMD myoblasts) express CAR at their surface. Direct fluorescence microcopy (Fig. 4A) and flow cytometry (Figs. 4B and 4C) showed a clear dose-dependent increase in gene transfer activity in both cell types. Moreover, flow cytometric quantification of the frequency of eGFP-positive cells (Fig. 4C, left graphs) and the average eGFP level per transduced cell (Fig. 4C, right graphs) revealed that, for each given vector dose, dHV.F50 and dHV.68/5′3′.F50 gave similar transduction levels.

**Figure 5:**
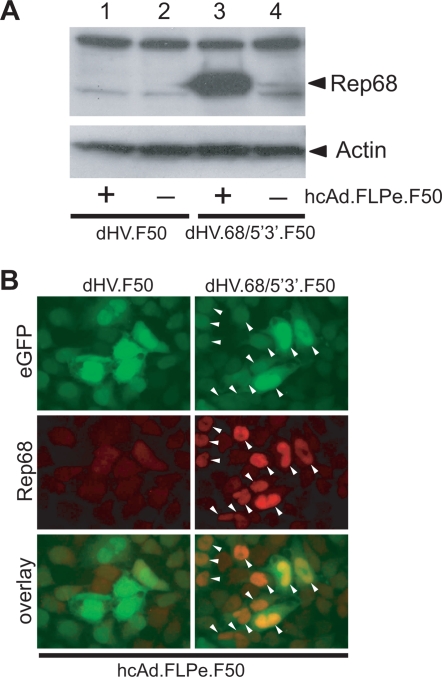
Testing the inducible *rep68* expression unit in dHV.68/5′3′.F50 particles purified by CsCl density gradient ultracentrifugation. (A) Western blot analysis of FLPe-induced Rep68 synthesis. HeLa cells were co-transduced with dHV.F50 and hcAd.FLPe.F50 (lane 1) or with dHV.68/5′3′.F50 and hcAd.FLPe.F50 (lane 3) or were transduced exclusively with dHV.F50 (lane 2) or with dHV.68/5′3′.F50 (lane 4). Three days postinfection, cellular proteins were extracted, separated in an SDS-10% polyacrylamide gel and transferred to a polyvinylidene fluoride membrane. Next, the membrane was incubated with a monoclonal antibody specific for all four AAV *rep* gene products. An antibody directed against actin served as a loading control. (B) eGFP direct fluorescence microscopy and Rep68 immunofluorescence microscopy on HeLa cells infected with hcAd.FLPe.F50 (50 GSAUs/cell) and either dHV.F50 (5 HTUs/cell; left column) or dHV.68/5′3′.F50 (5 HTUs/cell; right column). The pictures are derived from representative microscopic fields. The fluorescent signals derived from the green and red channels are overlaid in the lower panels. The arrowheads in the right panels point to cells containing both eGFP and Rep68. Note that the former protein is distributed throughout the cell whereas the latter is confined to the nucleus. Original magnification: 400×.

To investigate whether dHV.68/5′3′.F50 particles contain FLP-responsive *rep68*-specific gene switches, we performed Western blot analysis on lysates derived from HeLa cells transduced with CsCl density gradient-purified dHV.F50 and dHV.68/5′3′.F50 particles using a monoclonal antibody recognizing all four AAV *rep* gene products. HeLa cells were infected with dHV.F50 or dHV.68/5′3′.F50 alone (Fig. 5A; lanes 2 and 4) or in combination with hcAd.FLPe.F50 (Fig. 5A; lanes 1 and 3). Co-infection of the target cells with dHV.68/5′3′.F50 and hcAd.FLPe.F50 resulted in robust induction of *rep68* expression (Fig. 5A; lane 3). In contrast, the amount of Rep68 produced in dHV.68/5′3′.F50-infected HeLa cells in the absence of FLPe was very low (Fig. 5A; lane 4). These results demonstrate dHV.68/5′3′.F50-mediated transfer of functional *rep68* gene switch modules into target cells. Subsequently, to examine the synthesis of Rep molecules at the single-cell level we carried out eGFP direct fluorescence microscopy and Rep68 immunofluorescence microscopy on HeLa cells co-transduced with dHV.F50 and hcAd.FLPe.F50 (Fig. 5B; left panels) or with dHV.68/5′3′.F50 and hcAd.FLPe.F50 (Fig. 5B; right panels). The cells infected with the *rep68*-negative dHV served to establish background levels for the Rep68 immunostainings. Despite the possibility that not all target cells were transduced simultaneously by both vector types, the overlay of the eGFP- and Rep68-specific signals (Fig. 5B; lower row) demonstrated co-synthesis of both proteins in most of the cells exposed to dHV.68/5′3′.F50 and hcAd.FLPe.F50. As expected, eGFP located in the cytoplasm and in the nucleus whereas Rep68 was present in the latter compartment. Moreover, generally, a direct correlation between the strength of the eGFP- and the Rep68-specific signals was observed.

### Permanent genetic modification of HeLa cells and myoblasts from a DMD patient by rep68-positive and fiber-modified dHV particles

Finally, we tested the ability of dHV.68/5′3′.F50 to permanently transduce target cells by locus-specific transgene integration using two different human cell types, i.e., HeLa cells and DMD myoblasts. HeLa cells were chosen for this purpose because they constitute a reference cell line on which both wild-type AAV and Rep78/68-dependent DNA integration have been evaluated both in terms of stable transduction levels and targeted DNA insertion proficiencies. Myoblasts are being deployed in clinical trials for more than a decade to develop an allogeneic cell therapy for DMD [30]. One of the problems associated with this treatment modality is that it depends on strong and durable immunosuppression to prevent graft rejection. The use of gene-corrected autologous myoblasts may obviate the need to severely immunosuppress the patients. We previously showed that dystrophin-defective myoblasts are very well transduced *ex vivo* by non-integrating hcAd-based vectors carrying the receptor-interacting domains of hAd serotype 50 (hAd50) fibers [11]. This makes it relevant to test the ability of dHV.68/5′3′.F50 to stably transduce DMD myoblasts through targeted transgene integration. Moreover, contrary to HeLa cells, the DMD myoblasts used in this study possess a normal, diploid karyotype [31].

HeLa cells and DMD myoblasts were independently exposed to equal doses of dHV.F50 or dHV.68/5′3′.F50, either alone or together with a constant amount of hcAd.FLPe.F50. Three days later the initial levels of target cell transduction were determined by eGFP-directed flow cytometry. The frequencies of eGFP-positive cells were within a relatively narrow range, i.e., between 72 and 78% for the HeLa cells and between 76 and 81% for the DMD myoblasts. Next, the stable transduction frequencies (STFs) reached by dHV.F50 and by dHV.68/5′3′.F50 were determined by extensively subculturing both types of dividing cells and by measuring the percentages of eGFP-positive cells through flow cytometry as a function of time. This experimental setup does not introduce any bias towards the selection of cells carrying chromosomally integrated vector DNA and prevents that cells containing only episomal vector genomes are included in the STF calculations, which is a concern given the relatively long half-life of eGFP [32]. Stable transduction levels were reached at 27 days postinfection in HeLa cells whereas it took 56 days before the percentage of eGFP-positive DMD myoblasts stabilized, most likely reflecting the lower division rate of the latter cell type. The STFs obtained with dHV.68/5′3′.F50 in the presence of hcAd.FLPe.F50 were 0.6% (*n* = 2) and 0.4% (*n* = 1) for HeLa cells and DMD myoblasts, respectively (*n* events acquired = 10,000). In the absence of FLPe, dHV.68/5′3′.F50 yielded an STF of 0.05% (*n* = 2) for HeLa cells and of 0.1% (*n* = 1) for DMD myoblasts (*n* events acquired = 10,000). Thus, *rep68* gene activation increased by 12- and 4-fold the STF of dHV.68/5′3′.F50-transduced HeLa cells and DMD myoblasts, respectively. Importantly, infection of both target cell types with the control vector dHV.F50 alone or together with hcAd.FLPe.F50 gave rise to similar STFs as observed for HeLa cells and DMD myoblasts transduced exclusively with dHV.68/5′3′.F50.

**Figure 6:**
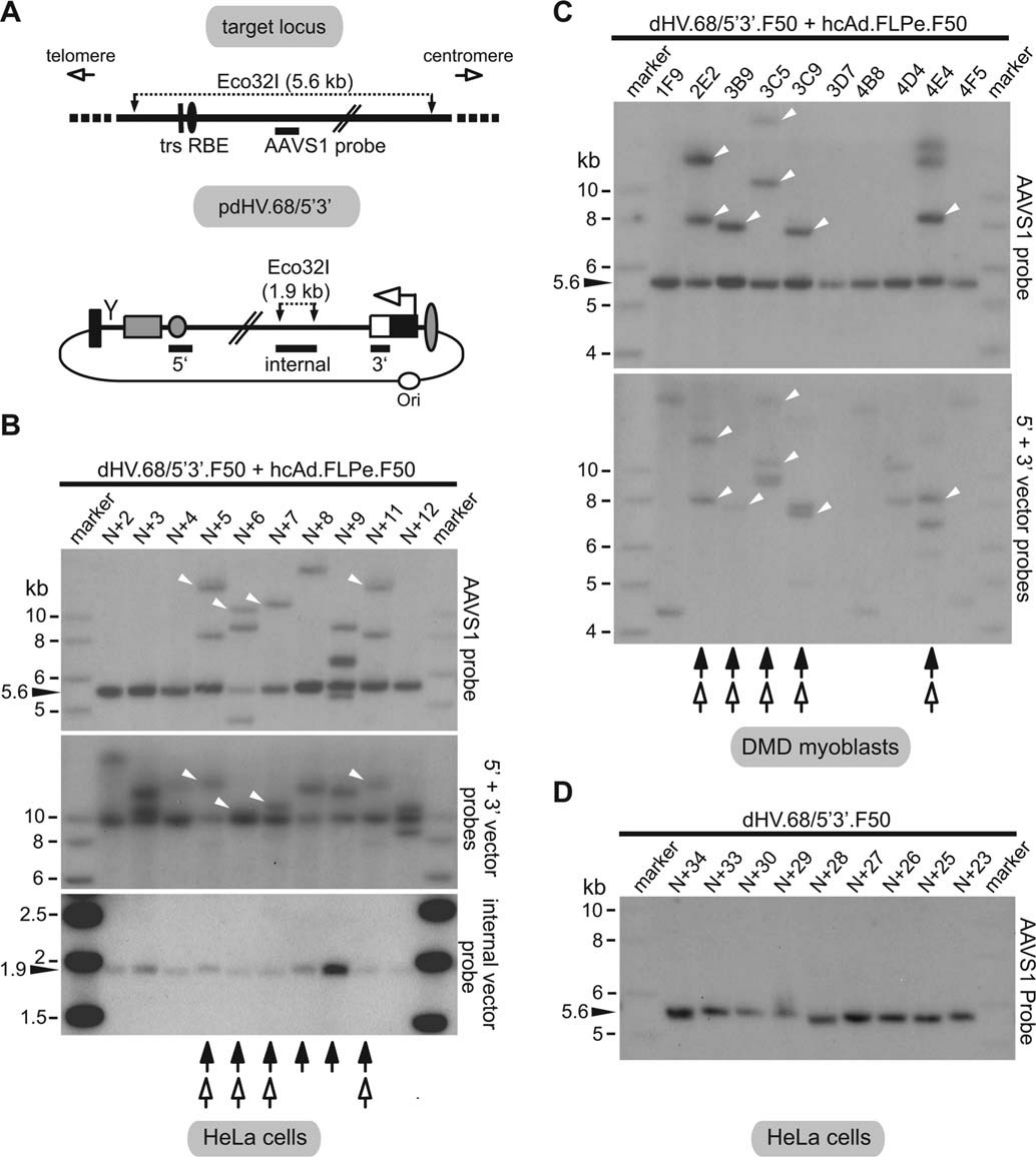
Examination of targeted versus random dHV.68/5′3′.F50 DNA insertion through Southern blot analysis of genomic DNA. (A) Upper panel, Eco32I restriction map of the AAV DNA preintegration region (i.e. the *AAVS1* locus) on human chromosome 19 at 19q13.3-qter. Vertical line and solid oval, trs and RBE, respectively. The vertical solid arrows indicate Eco32I recognition sites delimiting a 5.6-kb DNA segment. The solid horizontal bar denotes the *AAVS1*-specific probe drawn in relation to its target sequence (diagram not on scale). Lower panel, Eco32I restriction map of dHV.68/5′3′.F50 DNA showing the various hybridization probes (horizontal solid bars) drawn in relation to their cognate target sequences (drawing not on scale). For an explanation of symbols and abbreviations, see the legend of Fig. 1. (B) Ten micrograms of total cellular DNA from 10 representative clones derived from HeLa cells co-infected with dHV.68/5′3′.F50 and hcAd.FLPe.F50 were digested with Eco32I. Following agarose gel electrophoresis, the resolved DNA fragments were subjected to Southern blot analysis with the *AAVS1*-specific probe (*AAVS1* probe; upper panel). After removal of the *AAVS1*-specific probe, the transferred DNA was incubated simultaneously with a probe derived from the AAV p5IEE plus the *rβG* gene pA signal and a probe specific for the *eGFP* ORF (5′ and 3′ vector probes; middle panel). Finally, following a second stripping step, the transferred DNA was exposed to a fourth probe corresponding to the 1.9-kb Eco32I fragment in the dHV.68/5′3′.F50 genome (internal vector probe; lower panel). Clones derived from HeLa cells with AAV Rep68-induced disruptions in the *AAVS1* locus are indicated by solid arrows, whereas those that also have dHV.68/5′3′.F50 DNA inserted into this specific region of human chromosome 19 are discriminated by open arrows. Marker, GeneRuler DNA Ladder Mix molecular weight marker. (C) Ten micrograms of total cellular DNA from 10 clones of DMD myoblasts co-infected with dHV.68/5′3′.F50 and hcAd.FLPe.F50 were digested with Eco32I. Following agarose gel electrophoresis, the resolved DNA species were subjected to Southern blot analysis with the *AAVS1*-specific probe (*AAVS1* probe; upper panel) and, after its removal, with the two probes binding close to the termini of the dHV.68/5′3′.F50 genome (5′ and 3′ vector-specific probes; lower panel). Clones derived from DMD myoblasts with AAV Rep68-induced disruptions in the *AAVS1* locus are indicated by solid arrows, whereas those that also have dHV.68/5′3′.F50 DNA inserted into this specific region of human chromosome 19 are discriminated by open arrows. Marker, GeneRuler DNA Ladder Mix molecular weight marker. (D) Ten micrograms of total cellular DNA from 9 representative clones derived from HeLa cells infected with dHV.68/5′3′.F50 alone were digested with Eco32I. Following agarose gel electrophoresis, the resolved DNA fragments were subjected to Southern blot analysis with the *AAVS1*-specific probe. Marker, GeneRuler DNA Ladder Mix molecular weight marker.

To determine the frequencies of targeted DNA integration, stably transduced HeLa cells and DMD myoblasts were isolated from cell cultures initially exposed to both dHV.68/5′3′.F50 and hcAd.FLPe.F50 and individually expanded. HeLa cell clones derived from cultures infected exclusively with dHV.68/5′3′.F50 served as controls. Genomic DNA was extracted from the various cell lines and subjected to Eco32I digestion and Southern blot analysis. The transferred DNA was incubated first with an *AAVS1*-specific probe (Fig. 6A; upper panel) and, after membrane stripping, was exposed to a mixture of two probes binding immediately downstream of the *rep68* gene and close to the right end of the monomeric vector genome (Fig. 6A; lower panel). Examples of autoradiograms derived from these experiments are shown in Fig. 6B (HeLa cell clones) and Fig. 6C (DMD myoblast clones). Hybridization of a particular Eco32I fragment with both the *AAVS1*- and dHV.68/5′3′.F50-specific probes signifies insertion of vector DNA into the *AAVS1* locus on human chromosome 19 (Figs. 6B and 6C, open arrowheads). Subsequently, the membrane was once again stripped and incubated with a probe specific for a 1.9-kb Eco32I fragment in the vector genome (Fig. 6A; lower panel). The autoradiograms corresponding to hybridizations using the *AAVS1*-specific probe showed that 50% of the 38 randomly selected HeLa cell clones yielded DNA fragments with larger and, less often, smaller sizes than that of the 5.6-kb Eco32I fragment characteristic for undisrupted *AAVS1* alleles (Fig. 6B; upper panel and Table 1). This observation is consistent with the well-known capacity of Rep78 and/or Rep68 to locally amplify the *AAVS1* region due to the usage of its RBE and trs as an origin of replication [33]. Thus, the absence of detectable changes to the *AAVS1* loci in the other 50% of the HeLa cell clones most likely reflects the presence of no or few Rep68 molecules in their founder cells probably due to a lack of FLPe. In agreement with this interpretation, no disruptions of the *AAVS1* alleles were found after Southern blot analysis of chromosomal DNA extracted from 40 randomly selected clones of HeLa cells exposed exclusively to dHV.68/5′3′.F50 (Fig. 6D; Table 1), indicating that although “leaky” *rep68* expression may occur (Fig. 5A) in most cases the amount of Rep68 does not suffice to amplify the *AAVS1* region. Consistent with this finding, Southern blot hybridization using the vector-specific probes revealed that these clones did not have dHV.68/5′3′.F50 DNA integrated into the *AAVS1* region (data not shown). Conversely, in the presence of the FLPe-encoding hcAd vector, integration of dHV.68/5′3′.F50 DNA into the *AAVS1* locus could be demonstrated (Fig. 6B; middle panel and Table 1). The targeted DNA integration efficiency, as determined on the basis of the total number of HeLa cell clones analyzed was 24% (9/38), whereas if one considers exclusively clones with discernible changes in the *AAVS1* locus (as a surrogate indicator for cells that originally contained enough Rep68) it reached 47% (9/19; Fig. 6B; Table 1). The same analysis applied to autoradiograms corresponding to DMD myoblast clones revealed that these cells were targeted at the *AAVS1* locus with an efficient of 28% (5/18) or 100% (5/5; Fig. 6C; Table 1), respectively.

**Table 1:** Frequencies of targeted foreign DNA integration events derived from Southern blot analysis of dHV.68/5′3′.F50-transduced HeLa cell and DMD myoblast clones.

	HeLa cells		DMD myoblasts
	dHV.68/5′3′.F50−hcAd.FLPe.F50	dHV.68/5′3′.F50+hcAd.FLPe.F50	dHV.68/5′3′.F50+hcAd.FLPe.F50
Randomly selected eGFP^+^ clones (N)	40	38	18
Clones with AAVS1 disruptions (D)/N [%]	0/40 [0]	19/38 [50]	5/18 [28]
Targeted integration events/N [%]	0/40 [0]	9/38 [24]	5/18 [28]
Targeted integration events/D [%]	0/0 [0]	9/19 [47]	5/5 [100]

### Molecular characterization of AAVS1-foreign DNA junctions following dHV.68/5′3′.F50-mediated gene transfer

**Figure 7:**
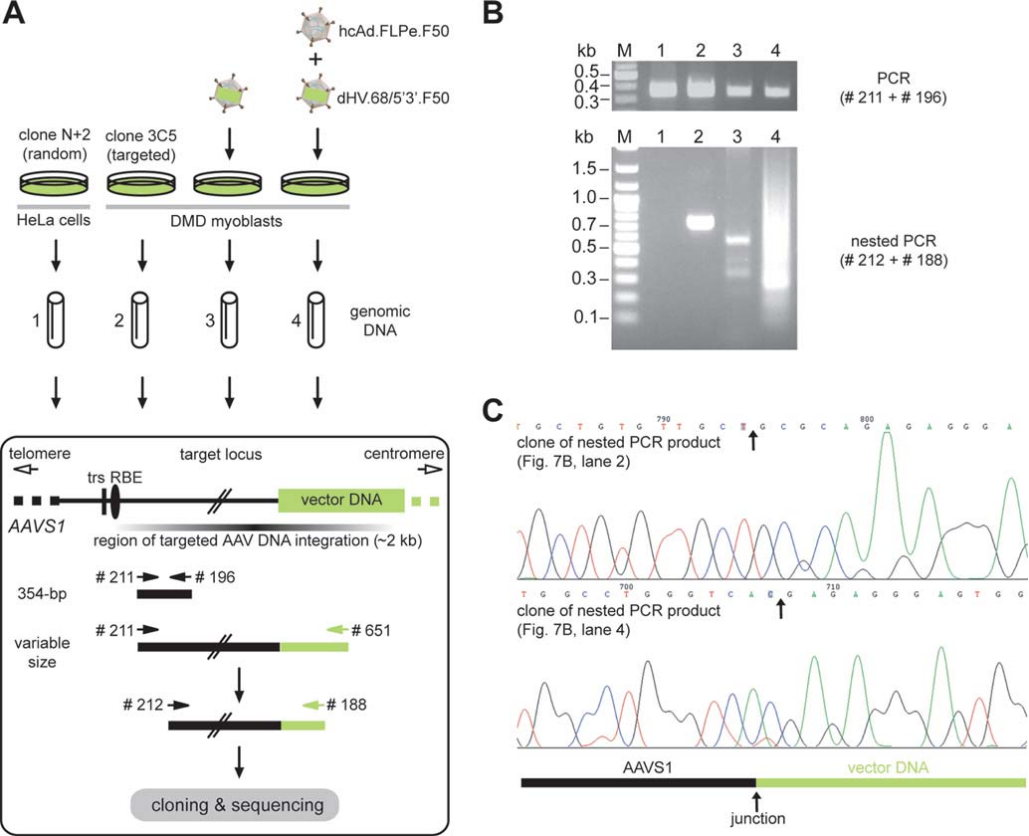
Integration of exogenous DNA into *AAVS1* after gene transfer with *rep68*-positive dHV.68/5′3′.F50 particles. (A) Overview of the PCR-based targeted DNA integration assay. Genomic DNA extracted from HeLa cell clone N+2 and DMD myoblast clone 3C5 served as control templates derived from cells genetically modified through vector DNA insertion outside and inside the *AAVS1* locus, respectively. The experimental samples consisted of chromosomal DNA from DMD myoblasts infected with dHV.68/5′3′.F50 alone (40 HTUs/cell) or co-infected with dHV.68/5′3′.F50 (40 HTUs/cell) and hcAd.FLPe.F50 (40 GSAUs/cell). The upper part of the boxed pictogram represents the section of human chromosome 19 at 19q13.3-qter harboring the *AAVS1* locus. Vertical line and adjacent oval, trs and RBE, respectively; horizontal fading bar, AAV DNA preintegration region; green box, representation of a targeted vector DNA insertion event leading to an *AAVS1*-foreign DNA junction; numbered black and green arrows symbolize *AAVS1*- and vector DNA-specific primers, respectively. (B) Agarose gel electrophoresis of PCR products resulting from amplifications carried out on chromosomal DNA from HeLa cell clone N+2 (lanes 1), DMD myoblast clone 3C5 (lanes 2), DMD myoblast population transduced with dHV.68/5′3′.F50 alone (lanes 3) and DMD myoblast population co-transduced with dHV.68/5′3′.F50 and hcAd.FLPe.F50 (lanes 4). Lanes M, Gene Ruler DNA Ladder Mix molecular weight marker. Upper panel, internal control PCR products resulting from amplifications performed with the *AAVS1*-specific primers # 211 and # 196. Lower panel, nested PCR products of amplifications carried out with oligodeoxyribonucleotides # 212 and # 188 on DNA synthesized with the aid of primers # 211 and # 651. (C) Nucleotide sequence analysis of junctions generated between *AAVS1* and exogenous DNA following co-transduction of DMD myoblasts with dHV.68/5′3′.F50 and hcAd.FLPe.F50. The DNA sequencing data in the upper and lower panel correspond to inserts derived from PCR amplifications on chromosomal DNA from myoblast clone 3C5 and from the DMD myoblast population co-transduced with dHV.68/5′3′.F50, respectively. The black and green bars indicate *AAVS1* and vector DNA, respectively. Vertical arrows points to the junction between *AAVS1* and dHV.68/5′3′.F50 DNA present in each individual clone.

To confirm by an independent method the ability of *rep68*-positive dHV particles to accomplish locus-specific foreign DNA insertion, we performed experiments aiming at the isolation of junctions between *AAVS1* and vector DNA. To this end, DMD myoblasts were transduced with dHV.68/5′3′.F50 alone or together with hcAd.FLPe.F50. Subsequently, chromosomal DNA was extracted from the transduced cells and *AAVS1*-vector DNA junctions were analyzed by PCR (Fig. 7A). Chromosomal DNA derived from human cells stably transduced by dHV.68/5′3′.F50 DNA in a non-targeted (i.e. HeLa cell clone N+2) and *AAVS1*-specific (i.e. DMD myoblast clone 3C5) manner served as negative and positive controls, respectively. The DNA derived from the DMD myoblast clone yielded a discrete PCR fragment whereas no amplification product was obtained with the DNA from the HeLa cell clone (Fig. 7B, lower panel, compare lanes 1 and 2). Nested PCR products were also detected in samples corresponding to reactions performed on DNA from DMD myoblast cultures transduced with dHV.68/5′3′.F50 alone (Fig. 7B, lower panel, lane 3) or together with hcAd.FLPe.F50 (Fig. 7B, lower panel, lane 4). Importantly, however, a much broader range of PCR product sizes was present in the sample derived from the cells that had been exposed to both vectors (Fig. 7B, lower panel, compare lanes 3 and 4). These results indicate dHV.68/5′3′.F50 DNA integration into the *AAVS1* locus and, consistent with the Southern blot data (Fig. 6 and Table 1), show that targeted integration events are more frequent in cells in which *rep68* expression is up-regulated by FLPe than in cells in which the *rep68* gene switch is not activated. AAV Rep78/68-dependent DNA integration into the *AAVS1* locus is not site-specific in the strict sense since it does not take place at a single nucleotide position but within a relatively long stretch of DNA. As a result, *AAVS1*-exogenous DNA junctions generated through the AAV machinery are usually present as single copies within a population of stably transduced cells. Thus, the smear detected in Fig. 7B, lane 4 most likely reflects the amplification of a mixture of *AAVS1*-dHV.68.5′3′.F50 DNA junctions with various sizes as observed following wild-type AAV DNA integration. To investigate this, PCR products from the smear (Fig. 7B, lane 4) were inserted into the cloning vector pCR-Blunt II-TOPO. The PCR fragment corresponding to the DMD myoblast clone 3C5 (Fig. 7B, lane 2) was cloned in parallel. Plasmid DNA purified from randomly selected colonies was digested with EcoRI to release the inserts. As expected, agarose gel electrophoresis revealed differently sized inserts reflecting the variation in vector DNA insertion sites within the *AAVS1* locus (data not shown). Finally, DNA sequencing of these inserts representing individual integration events revealed the precise nucleotide location at which target site and vector DNA covalently joined each other. Two examples are depicted in Fig. 7C. Altogether, these experiments complement the Southern blot data presented in Fig. 6 and firmly establish the capacity of *rep68*-positive dHV particles to accomplish insertion of large DNA at a specific locus in the human genome.

## Discussion

The ability of wild-type AAV to insert its DNA into a specific genomic region in cultured cells of higher primates is unique among all known eukaryotic viruses. However, the AAV DNA integration mechanism remains sketchy, partially owing to its complexity [34]. For example, it is not known whether the pre-integration complexes consist of DNA molecules with linear, circular or both types of topologies. There is evidence, nonetheless, for genomic insertion through circular intermediates from studies showing (i) the capacity of rAAV plasmids to integrate into the *AAVS1* locus, (ii) the generation of circular double-stranded DNA via AAV ITR-mediated intermolecular recombination and (iii) the presence in cells latently infected with wild-type AAV of head-to-tail proviral tandem repeats [34]. The unique structure of dHV genomes consisting of linear DNA molecules that lack AAV ITRs at one or both ends in their monomeric and dimeric configuration, respectively, precludes the generation of circular integration substrates via AAV ITR-mediated recombination. Our finding that Rep68 can catalyze the insertion of dHV DNA into the *AAVS1* region thus suggests that linear templates are also amenable to the AAV Rep78/68-dependent integration process.

Although detailed knowledge about the AAV DNA integration mechanism is lacking, researchers are actively pursuing the development of gene delivery systems that exploit the desirable feature of locus-specific integration provided by AAV elements [4], [5], [35], [36]. When compared to non-targeted integrating vectors, these gene transfer systems might impose a lower risk of insertional oncogenesis caused by the inactivation of tumour suppressor genes or, as observed in clinical trials using gammaretroviral vectors, by the dysregulation of proto-oncogenes [37]. Among the many viral and non-viral vector systems capitalizing on the AAV integration machinery are those based on hcAd vectors [14], [38], [39]. Recchia and colleagues incorporated rAAV DNA encoding GFP and hygromycin B phosphotransferase into an hcAd vector genome [38]. Co-infection of Hep3B cells with the resulting vector and another hcAd vector containing a bacteriophage T7 promoter-driven *rep78* gene at a multiplicity of infection (MOI) of 10 transducing units per cell each followed by hygromycin B selection revealed that the overall STF was 0.4% (i.e. 400 hygromycin B-resistant clones/10^5^ target cells). According to the authors, similar results were obtained in other human hepatic cell lines [38]. Of note, the STF was not enhanced by AAV *rep78* expression. However, Rep proteins did promote the insertion of the AAV ITR-flanked DNA into the *AAVS1* locus in 14/39 (i.e. 35%) clones of stably transduced HepG2 cells [38]. Similar results were obtained with a single-particle version of this hcAd/AAV hybrid vector system accommodating a 3.8-kb rAAV genome and an inducible *rep78* expression unit regulated by a doxycycline-controlled transcriptional silencer [40]. Recently, Wang and Lieber generated capsid-modified hcAd vectors containing a GFP-encoding rAAV genome significantly larger than those tested by Recchia and co-workers [39]. In parallel, they succeeded in rescuing and propagating a Rep78-encoding hcAd vector by relying on a strategy based again on a transcriptional regulatory element with low basal activity in producer cells (i.e. the human β-globin locus control region). Co-infection of human megakaryoblastic leukemia (Mo7e) cells with the rAAV- and *rep78*-containing hcAd vectors at an MOI of 300 and 800 genomes per cell, respectively, followed by their clonal expansion yielded 25.8% cell clones containing stably transduced Mo7e cells. However, the percentage of GFP-positive cells in individual Mo7e clones varied greatly, which makes it very hard to accurately determine the actual STF. Nonetheless, from data presented in Fig. 9 of the study by Wang and Lieber [39] one can deduce that the STF at best was in the order of a few percent. Substitution of the *rep78*-positive for a *rep78*-negative hcAd vector resulted in somewhat lower stable transduction levels [39]. Finally, in 30% of the stably transduced clones derived from Mo7e cells co-infected with the rAAV- and *rep78*-containing hcAd vectors, the foreign DNA was found to be inserted into the *AAVS1* region [39].

In this study, we report the generation of tropism-modified dHVs containing AAV *rep68* expression units and show their ability to mediate integration of DNA fragments of at least 10.3 kb into the *AAVS1* locus on human chromosome 19 in HeLa and muscle progenitor cells. Side-by-side rescue and propagation experiments revealed that the growth properties of the *rep68*-positive dHVs are very similar to that of the *rep68*-negative control vector. These results signify that the amounts of Rep68 made during the rescue and propagation of the *rep68*-positive dHVs were not enough to markedly inhibit their production. Transduction of rapidly proliferating human cells with dHV.68/5′3′.F50 particles followed by the activation of the *rep68* gene switch incorporated in the vector genome led to the insertion at 19q13.3-qter of large DNA molecules that included a functional transgene expression unit and human dystrophin-coding sequences. Significantly, in HeLa cells exposed exclusively to dHV.68/5′3′.F50 (i.e. hcAd.FLPe.F50-negative cells), the basal Rep68 levels did not suffice to disrupt *AAVS1* loci and, in agreement with this observation, no targeted DNA insertion could be detected in any of the 40 clones that were analyzed (Fig. 6). However, by using a more sensitive PCR-based assay, evidence for low-level *AAVS1*-targeted vector DNA insertion in FLPe-negative cells was found (Fig. 7B, lane 3).

An interesting aspect of the present study is the side-by-side comparison of the performance of an AAV Rep78/68-dependent DNA targeting system in cell types with very different genetic stabilities. HeLa cells are notorious for their aneuploidy and widespread translocations, whereas the human myoblasts used in this study remain diploid even after many cell divisions [31]. If one considers only those cells in which Rep68 induced unambiguous *AAVS1* disruptions, the targeted DNA insertion efficiency reached 47% in HeLa cells (9/19 clones) and a remarkable 100% in DMD myoblasts (5/5 clones). It is thus tempting to speculate that the lower targeted DNA integration proficiency of hybrid vector genomes in HeLa cells is a consequence of the higher genetic instability of these tumor cells causing a substantial fraction of vector genomes to insert randomly at double-stranded breaks possibly through non-homologous end-joining as observed with *rep*-negative rAAV DNA [41]. This would suggest that in the presence of appropriate amounts of Rep78 and/or Rep68 molecules, dHV DNA might indeed display a marked preference for locus-specific insertion in the genomes of therapeutically relevant (i.e. non-transformed) cell types. Further experiments that might include tests on a larger panel of cell types are warranted to establish or disprove this postulate.

Importantly, we demonstrated that transduction of human cells with capsid-modified dHV particles can lead to long-term transgene expression in an AAV Rep68-dependent manner. However, notwithstanding the diverse experimental setups used, a common feature of this and other Rep78/68-dependent integrating hcAd vector-based gene carriers is their overall low STFs. The low STFs reached by these hybrid vector systems are at odds with those reported for wild-type AAV in human cells in culture. For instance, using Southern blot analyses, Hamilton and colleagues deduced that wild-type AAV can stably transduce up to 50% of infected HeLa cells [42]. In addition, by deploying a quantitative real-time PCR assay, other investigators showed that wild-type AAV DNA integrates at 19q13.3-qter in at least 15% of infected HeLa cells [43], [44]. Recchia and colleagues found for their monopartite hcAd/AAV hybrid vector system that the efficiency of *AAVS1*-specific integration was directly proportional to the Rep78 level in the target cells [40]. However, as mentioned previously, above a certain concentration, which might be different for different cell types, the two largest AAV Rep proteins have cytostatic or even cytotoxic properties. The low STFs achieved by the Rep78/68-dependent integrating hcAd vectors in long-term experiments deploying rapidly proliferating cells may thus relate to uncontrolled Rep78/68 synthesis. According to this hypothesis, many of the cells that have vector DNA integrated into the *AAVS1* locus will display a strong growth disadvantage due to (persistent) high Rep78/68 levels. This will result in the selection of cells that contain no or few Rep78/68 molecules and are therefore not stably transduced or have the vector DNA integrated outside of the *AAVS1* region.

The idea of a strong selection against cells displaying high Rep68 activity is consistent with the observation that although most cells that initially received dHV.68/5′3′.F50 were also transduced by hcAd.FLPe.F50, a significant proportion of the randomly isolated HeLa cell and DMD myoblast clones did not show evidence of Rep68-induced *AAVS1* disruptions (i.e., 50 and 72%, respectively). Future hcAd/AAV hybrid vector systems should therefore be designed in such a way as to ensure that the Rep78/68 activity is minimal in producer cells and high but short-lived in target cells. Inducible gene expression cassettes encoding Rep78 and/or Rep68 variants whose stability or subcellular localization can be controlled by cell-permeable small-molecule drugs may help to fulfill these requirements. Such controllable gene expression may, in addition, allow the production of *rep68*-positive dHV particles that are not dependent on the introduction of FLPe or on any other foreign protein into target cells.

## Materials and Methods

### Cells

Culturing conditions for the human cervical carcinoma (HeLa) cells (American Type Culture Collection), the hAd5 early region 1 (E1)-deleted and bacteriophage P1 *cre*-expressing PER.tTA.Cre76 cells and the DMD myoblasts have been described elsewhere [11], [31], [45].

### DNA constructions

Recombinant DNA techniques were performed by using established methods [46] or following the instructions supplied with specific reagents. Large-scale plasmid DNA purifications were carried out using JetStar 2.0 kits (Genomed) according to the manufacturer's recommendations. All DNA constructs as well as their nucleotide sequences are available upon request.

### Viral vectors

The production and titration of stocks of the E1-deleted and capsid-modified helper Ad vector Ad.floxedΨ.F50 have been previously described [11]. Ad.floxedΨ.F50 particles display at their surface chimeric fiber proteins consisting of basal shaft sequences from hAd5 and apical shaft and knob domains from hAd50 [11]. The rescue and propagation of dHV.F50, dHV.68/3′5′.F50 and dHV.68/5′3′.F50 were carried out essentially as described elsewhere [11], [14]. The titers of dHV.F50, dHV.68/3′5′.F50 and dHV.68/5′3′.F50 stocks were determined by flow cytometry [11], [14] on the basis of *eGFP* expression and are expressed as HeLa-cell transducing units (HTUs) per ml. The fiber-modified hcAd vector hcAd.FLPe.F50 served as source of FLPe [17]. hcAd.FLPe.F50 particles were produced in PER.tTA.Cre76 cells using the hcAd vector shuttle plasmid pAd.FLPe.dAgeI together with Ad.floxedΨ.F50. pAd.FLPe.dAgeI was derived from construct pAdFLPe [18] by removal of a 3.3-kb AgeI fragment comprising stuffer sequences derived from the human *hypoxanthine guanine phosphoribosyltransferase 1* gene. As a consequence, the FLPe-encoding hcAd.FLPe.F50 vector particles contain a 27.4-kb genome. Titration of the hcAd.FLPe.F50 preparation was performed on HeLa cells containing the FLP-responsive construct pGS.pA+.DsRed [28]. Induction of the synthesis of the fluorescent reporter protein DsRed.T4 was determined through flow cytometry and the corresponding functional titers were expressed as gene switch-activating units (GSAUs) per ml.

### Flow cytometry

Quantification of eGFP- or DsRed.T4-positive cells was carried out by using a BD LSR II flow cytometer (Becton Dickinson). In all instances, 10,000 events were acquired. Data was analysed with the aid of BD FACSDiva Software version 5.0.1 (Becton Dickinson).

### DNA transfections

Plasmid DNA transfections were carried out with Lipofectamine (Invitrogen) as specified before [14].

### AAV-dependent DNA replication assay

The functionality of the FLP-activatable AAV serotype 2 (AAV2) *rep68* expression plasmid pGS.pA+.Rep68 and of hcAd.FLPe.F50 were evaluated as follows. PER.tTA.Cre76 cells were seeded in 6-well plates (Greiner) at a density of 2.5×10^6^ cells per well. The next day, these cells were transfected with 1.5 µg of pGS.pA+.Rep68 and 1.5 µg of the rAAV vector shuttle construct pAAV.DsRed. pAAV.DsRed contains an expression unit consisting of a chimeric human *elongation factor 1α* (hEF1α) gene promoter [47], the *DsRed.T4* ORF and the rabbit *β-globin* (rβG) gene polyadenylation (pA) signal. The following day, the transfected cells were co-infected with Ad.floxedΨ.F50 (5 infectious units [IUs]/cell) and hcAd.FLPe.F50 (50 GSAUs/cell). PER.tTA.Cre76 cells transfected with 1.5 µg of pAAV.DsRed and 1.5 µg of the AAV2 *rep78/68* expression plasmid pGAPDH.Rep78/68 and infected with Ad.floxedΨ.F50 at an MOI of 5 IUs per cell served as positive control. pGAPDH.Rep78/68 directs the constitutive synthesis of Rep78 and Rep68 from the promoter of the human *glyceraldehyde-3-phosphate dehydrogenase* (hGAPDH) gene [14]. Negative controls consisted of pGS.pA+.Rep68- and pAAV.DsRed-transfected PER.tTA.Cre76 cells infected with Ad.floxedΨ.F50 alone and of pGAPDH.Rep78/68- and pAAV.DsRed-transfected PER.tTA.Cre76 cells that were not exposed to hcAd.FLPe.F50 or Ad.floxedΨ.F50. Three days posttransfection, the cells were harvested and processed for extrachromosomal DNA extraction.

### Extrachromosomal DNA extraction and analysis

The purification of extrachromosomal DNA was performed as previously described [48]. To selectively degrade the prokaryotic input DNA, the samples were treated for 1 h at 37°C with *Dpn*I. After agarose gel electrophoresis through 1% agarose gels in 1× Tris-acetate-EDTA buffer, the DNA was transferred by capillary action onto nylon membranes (Amersham Hybond-XL; GE Healthcare). These membranes were subsequently incubated with blocking solution, exposed to the DNA probe specified below and repeatedly washed using a standard Southern hybridization procedure. Finally, the Southern blots were exposed to autoradiography films (Amersham Hyperfilm MP; GE Healthcare).

rAAV-specific replication products were detected with the aid of the 0.7-kb NcoI×Eco52I fragment from pAAV.DsRed, which includes the entire *DsRed.T4* ORF. This DNA probe was labeled with [α-^32^P]dATP (GE Healthcare) by using the HexaLabel DNA Labeling Kit (Fermentas).

### Transduction experiments

HeLa cells and DMD myoblasts were seeded at densities of 1.0×10^5^ and 4.0×10^5^ cells per well of 24-well plates (Greiner), respectively. After an overnight incubation period the cells were exposed for 24 hours to dHV.50 and dHV.68/5′3′.F50 at 4, 20 and 100 HTUs/cell in a total volume of 500 µl. Direct fluorescence microscopy and eGFP-directed flow cytometry were carried out 72 hours postinfection. Mock-infected HeLa cells and DMD myoblasts processed in parallel served to set the background levels of each assay.

### Light microscopy and immunostaining

eGFP direct fluorescence microscopy and AAV Rep immunofluorescence microscopy were performed with an Olympus IX51 inverse fluorescence microscope. Images were captured by a ColorView II Peltier-cooled charge-coupled device camera and were archived using analySIS software (Soft Imaging Systems). Cells were processed for immunostaining as described before [11] except that the primary antibody solution consisted of a 1∶50 dilution of murine monoclonal antibody 303.9 (PROGEN Biotechnik; catalog number: 61069), which recognizes all four AAV Rep proteins.

### Western blot analysis

Two-cm^2^ monolayers of HeLa cells were washed once with phosphate-buffered saline and dissolved in 100 µl ice-cold lysis buffer consisting of 20 mM Tris-HCl (pH 7.6), 150 mM NaCl, 0.1% sodium dodecyl sulfate (SDS), 0.5% sodium deoxycholate, 1% Nonidet P40 and 10% glycerol plus a cocktail of protease inhibitors (Complete Mini, Roche Applied Science). The protein lysates were spun for 10 min at 4°C in a minicentrifuge at 25,000× *g*. Fifteen µl of the resulting supernatants were mixed with 3 µl of 6× sample buffer (360 mM Tris-HCl [pH 6.7], 60% glycerol and 12% SDS), which was supplemented immediately prior to use with 150 µl of β-mercaptoethanol and 150 µl of a saturated bromophenol blue solution per 2.5 ml. Next, the samples were heated for 5 min at 100°C and subjected to electrophoresis through an SDS-10% polyacrylamide gel. Resolved proteins were subsequently transferred onto an Immobilon-P membrane (Millipore) by electroblotting. After incubation for 2 h at room temperature (RT) with blocking solution (10 mM Tris-HCl [pH 8.0], 150 mM NaCl and 0.05% Tween-20 [TBST]) containing 10% non-fat dry milk powder (Elk, Campina), the Western blot was incubated overnight at 4°C with monoclonal antibody 303.9 diluted 1∶50 in blocking solution. Next, the Immobilon membrane was washed 3 times for 5 min each with TBST at RT and subjected to a 1-h incubation with horseradish peroxidase-conjugated goat-anti-mouse IgG secondary antibodies (Santa Cruz Biotechnoloy; catalog number: sc-2005) diluted 1∶10,000 in blocking solution. After three 20-min washes with TBST, the Western blot was incubated for 5 min with chemiluminescence substrate solution (100 mM Tris-HCl [pH 8.6], 250 µg/ml 5-amino-2,3-dihydro-1,4-phthalazinedione [Sigma-Aldrich]) which was supplemented immediately prior to use with 20 µl of a 30% H_2_O_2_ solution and 1 ml of a 0.33% 3-(4-hydroxyphenyl)-2-propenoic acid (Sigma-Aldrich) solution in dimethylsulfoxide per 100 ml. The chemiluminescent signal was visualized by exposing the Immobilon membrane to an Amersham Hyperfilm MP film. Actin served as loading control and was detected by using a 1∶5,000 dilution of a mouse monoclonal antibody (clone: C4; MP Biomedicals; catalog number: 691001).

### Chromosomal DNA extraction and analysis

The purification of chromosomal DNA was carried essentially as previously described [49] except for the use of a different lysis buffer consisting of 100 mM Tris-HCl (pH 8.5), 5 mM EDTA (pH 8.0), 0.2% SDS and 200 mM NaCl. After restriction enzyme digestion with Eco32I (Fermentas) and electrophoresis through 0.8% agarose gels in 1× Tris-acetate-EDTA buffer, the DNA was transferred by capillary action onto Amersham Hybond-XL membranes. These membranes were subsequently incubated with blocking solution, exposed to the DNA probes specified below and repeatedly washed using a standard Southern hybridization procedure. Next, the Southern blots were exposed to autoradiography films. Before incubating the nylon membranes with new probes, the previous probes were removed by soaking them in a boiling 0.1% SDS solution. After cooling to RT the 0.1% SDS solution was discarded and the procedure was repeated twice.

The *AAVS1*-specific probe (353 bp) was obtained by PCR amplification using the template, primer pair and cycling parameters described elsewhere [14]. The 377-bp probe covering the AAV p5 promoter and *rβG* gene pA signal was also generated by PCR using construct pAd/AAV.EGFP [14] as template and the oligodeoxyribonucleotides 5′-GTCCATTCCTTATTCCATAGAAAAGC-3′ plus 5′-CCATCGATTGGAGTCGTGACGTGAATTACGTCATAGGGTTAGGGAGGTCCTGTATTAGAGGTCACG-3′,whereas the *eGFP*-specific probe (596 bp) was PCR amplified from plasmid pEGFP-N1 (Clontech) with the aid of the primers 5′-GAGCTGGACGGCGACGTAAACG-3′ and 5′-CGCTTCTCGTTGGGGTCTTTGCT-3′. The final concentrations of template, primers (Invitrogen), dNTPs (Fermentas) and GoTaq polymerase (Promega) in the 50-µl PCR mixtures were 20 pg/µl, 0.2 µM, 0.2 mM and 50 mU/µl, respectively. All PCR-derived probes as well as the 1.9-kb human *dystrophin* cDNA-specific Eco32I fragment used as an internal vector probe were purified by agarose gel electrophoresis followed by gel extraction using the QIAEX II system (Qiagen) according to the instructions provided by the manufacturer. Prior to their addition to the hybridization solution, the radiolabeled DNA probes were separated from unincorporated dNTPs through size-exclusion chromatography by using Sephadex-50 (GE Healthcare) columns.

### PCR-based targeted DNA integration assay

Four hundred thousand DMD myoblasts were seeded in wells of 24-well plates. After overnight incubation, the cells were infected with dHV.68/5′3′.F50 alone (40 HTUs/cell) or were co-infected with dHV.68/5′3′.F50 (40 HTUs/cell) and hcAd.FLPe.F50 (40 GSAUs/cell). The cells were exposed to the viral vectors for 24 hours after which the inocula were substituted by fresh culture medium. At 72 hours postinfection, genomic DNA was extracted from these cells as described above. Chromosomal DNA was also extracted from HeLa cell clone N+2 and DMD myoblast clone 3C5, which were derived from cells stably transduced with dHV.68/5′3′.F50 DNA through random and *AAVS1*-targeted DNA insertion, respectively. Next, samples containing 160 ng of genomic DNA were subjected to PCR with the *AAVS1*-specific primer # 211 (5′-CAGGTCCACCCTCTGCTG-3′), together with primer # 651 (5′-TTCCTAACCCCAACACTTGC-3′) targeting the vector DNA (i.e. *hEF1α* promoter sequence). PCR amplifications with the *AAVS1*-specific oligodeoxyribonucleotides # 211 and # 196 (5′-GGCTCAACATCGGAAGAGG-3′) were carried out in parallel to serve as a control for template integrity and are expected to give rise to a 354-bp PCR product. PCR mixtures of 50 µl containing 200 µM dNTPs, 0.2 µM of each primer, 0.012 U/µl of Phusion High-Fidelity DNA polymerase (Finnzymes) and 1× GC buffer (Finnzymes) were placed in a DNA Engine Tetrad 2 thermal cycler (Bio-Rad) and a touchdown PCR program was initiated by a 3-min incubation at 98°C, followed by 19 cycles of 98°C for 20 sec, an annealing period of 30 sec with the temperature decreasing by 0.5°C every cycle from 62 to 52°C, and a 2-min extension step at 72°C. When the PCR program reached the lower annealing temperature of 52°C, 28 additional cycles were performed by using the same cycling conditions except for the use of a constant annealing temperature of 52°C. This was followed by a 5-min incubation at 72°C. Next, 1 µl of each PCR sample was subjected to a nested PCR with the *AAVS1*- and vector DNA-specific primers #212 (5′-GCTTTGCCACCCTATGCTGAC-3′) and # 188 (5′-GGATCTGAGGAACCCCTAGTGATGG-3′), respectively. The cycling conditions applied to the nested PCR were as described above except for the use of a maximal annealing temperature of 69°C and a final touchdown temperature of 59°C. Samples from all PCR amplifications were subjected to agarose gel electrophoresis. Subsequently, PCR products were purified from the gel with the aid of the JetSorb gel extraction kit (GENOMED) and the recovered DNA was inserted into cloning vector pCR-Blunt II-TOPO (Invitrogen) according to the instructions supplied by the manufacturers. Finally, the nucleotide sequence of several of the cloned PCR fragments was determined as previously described [45] by using the M13 forward (5′-GACGTTGTAAAACGACGGCCAGT-3′) or reverse (5′-CAGGAAACAGCTATGACCATGA-3′) primer.

## References

[1] Kochanek S, Schiedner G, Volpers C (2001). High-capacity ‘gutless’ adenoviral vectors.. Curr Opin Mol Ther.

[2] Palmer DJ, Ng P (2005). Helper-dependent adenoviral vectors for gene therapy.. Hum. Gene Ther.

[3] Gonçalves MAFV, de Vries AAF (2006). Adenovirus: from foe to friend.. Rev Med Virol.

[4] Mitani K, Kubo S (2002). Adenovirus as an integrating vector.. Curr Gene Ther.

[5] Jager L, Ehrhardt A (2007). Emerging adenoviral vectors for stable correction of genetic disorders.. Curr Gene Ther.

[6] Gonçalves MAFV (2005). Adeno-associated virus: from defective virus to effective vector.. Virol J.

[7] Philpott NJ, Gomos J, Berns KI, Falck-Pedersen E (2002). A p5 integration efficiency element mediates Rep-dependent integration into AAVS1 at chromosome 19.. Proc Natl Acad Sci USA.

[8] Shayakhmetov DM, Papayannopoulou T, Stamatoyannopoulos G, Lieber A (2000). Efficient gene transfer into human CD34^+^ cells by a retargeted adenovirus vector.. J Virol.

[9] Knaän-Shanzer S, van der Velde I, Havenga MJE, Lemckert AAC, de Vries AAF (2001). Highly efficient targeted transduction of undifferentiated human hematopoietic cells by adenoviral vectors displaying fiber knobs of subgroup B.. Hum Gene Ther.

[10] Knaän-Shanzer S, van de Watering MJM, van der Velde I, Gonçalves MAFV, Valerio D (2005). Endowing human adenovirus serotype 5 vectors with fiber domains of species B greatly enhances gene transfer into human mesenchymal stem cells.. Stem Cells.

[11] Gonçalves MAFV, Holkers M, Cudré-Mauroux C, van Nierop GP, Knaän-Shanzer S (2006). Transduction of myogenic cells by retargeted dual high-capacity hybrid viral vectors: robust dystrophin synthesis in duchenne muscular dystrophy muscle cells.. Mol Ther.

[12] Stecher H, Shayakhmetov DM, Stamatoyannopoulos G, Lieber A (2001). A capsid-modified adenovirus vector devoid of all viral genes: assessment of transduction and toxicity in human hematopoietic cells.. Mol Ther.

[13] Balamotis MA, Huang K, Mitani K (2004). Efficient delivery and stable gene expression in a hematopoietic cell line using chimeric serotype 35 fiber pseudotyped helper-dependent adenoviral vector.. Virology.

[14] Gonçalves MAFV, van Nierop GP, Tijssen MR, Lefesvre P, Knaän-Shanzer S (2005). Transfer of the full-length dystrophin-coding sequence into muscle cells by a dual high-capacity hybrid viral vector with site-specific integration ability.. J Virol.

[15] Weitzman MD, Fisher KJ, Wilson JM (1996). Recruitment of wild-type and recombinant adeno-associated virus into adenovirus replication centers.. J Virol.

[16] Timpe JM, Verrill KC, Trempe JP (2006). Effects of adeno-associated virus on adenovirus replication and gene expression during coinfection.. J Virol.

[17] Buchholz F, Angrand PO, Stewart AF (1998). Improved properties of FLP recombinase evolved by cycling mutagenesis.. Nat Biotechnol.

[18] Kreppel F, Kochanek S (2004). Long-term transgene expression in proliferating cells mediated by episomally maintained high-capacity adenovirus vectors.. J Virol.

[19] Zhang HG, Wang YM, Xie JF, Liang X, Hsu HC (2001). Recombinant adenovirus expressing adeno-associated virus cap and rep proteins supports production of high-titer recombinant adeno-associated virus.. Gene Ther.

[20] Steinwaerder DS, Carlson CA, Lieber A (1999). Generation of adenovirus vectors devoid of all viral genes by recombination between inverted repeats.. J Virol.

[21] Carlson CA, Steinwaerder DS, Stecher H, Shayakhmetov DM, Lieber A (2002). Rearrangements in adenoviral genomes mediated by inverted repeats.. Methods Enzymol.

[22] Zhou C, Trempe JP (1999). Induction of apoptosis by cadmium and the adeno-associated virus Rep proteins.. Virology.

[23] Saudan P, Vlach J, Beard P (2000). Inhibition of S-phase progression by adeno-associated virus Rep78 protein is mediated by hypophosphorylated pRb.. EMBO J.

[24] Schmidt M, Afione S, Kotin RM (2000). Adeno-associated virus type 2 Rep78 induces apoptosis through caspase activation independently of p53.. J Virol.

[25] Giraud C, Winocour E, Berns KI (1995). Recombinant junctions formed by site-specific integration of adeno-associated virus into an episome.. J Virol.

[26] Xiao X, Xiao W, Li J, Samulski RJ (1997). A novel 165-base-pair terminal repeat sequence is the sole cis requirement for the adeno-associated virus life cycle.. J Virol.

[27] Parks RJ, Graham FL (1997). A helper-dependent system for adenovirus vector production helps define a lower limit for efficient DNA packaging.. J Virol.

[28] Holkers M, de Vries AAF, Gonçalves MAFV (2006). Modular and excisable molecular switch for the induction of gene expression by the yeast FLP recombinase.. Biotechniques.

[29] Rinaudo D, Lamartina S, Roscilli G, Filiberto G, Toniatti C (2000). Conditional site-specific integration into human chromosome 19 by using a ligand-dependent chimeric adeno-associated virus/Rep protein.. J Virol.

[30] Skuk D, Tremblay JP (2003). Myoblast transplantation: the current status of a potential therapeutic tool for myopathies.. J Muscle Res Cell Motil.

[31] Cudré-Mauroux C, Occhiodoro T, König S, Salmon P, Bernheim L (2003). Lentivector-mediated transfer of Bmi-1 and telomerase in muscle satellite cells yields a duchenne myoblast cell line with long-term genotypic and phenotypic stability.. Hum Gene Ther.

[32] Corish P, Tyler-Smith C (1999). Attenuation of green fluorescent protein half-life in mammalian cells.. Protein Eng.

[33] Young SM, McCarty DM, Degtyareva N, Samulski RJ (2000). Roles of adeno-associated virus Rep protein and human chromosome 19 in site-specific recombination.. J Virol.

[34] McCarty DM, Young SM, Samulski RJ (2004). Integration of adeno-associated virus (AAV) and recombinant AAV vectors.. Annu Rev Genet.

[35] Glauser DL, Ackermann M, Saydam O, Fraefel C (2006). Chimeric herpes simplex virus/adeno-associated virus amplicon vectors.. Curr Gene Ther.

[36] Smith RH (2008). Adeno-associated virus integration: virus versus vectors.. Gene Ther. Epub ahead of print.

[37] Fischer A, Cavazzana-Calvo M (2005). Integration of retroviruses: a fine balance between efficiency and danger.. PLoS Med.

[38] Recchia A, Parks RJ, Lamartina S, Toniatti C, Pieroni L (1999). Site-specific integration mediated by a hybrid adenovirus/adeno-associated virus vector.. Proc Natl Acad Sci USA.

[39] Wang H, Lieber A (2006). A helper-dependent capsid-modified adenovirus vector expressing adeno-associated virus rep78 mediates site-specific integration of a 27-kilobase transgene cassette.. J Virol.

[40] Recchia A, Perani L, Sartori D, Olgiati C, Mavilio F (2004). Site-specific integration of functional transgenes into the human genome by adeno/AAV hybrid vectors.. Mol Ther.

[41] Miller DG, Petek LM, Russell DW (2004). Adeno-associated virus vectors integrate at chromosome breakage sites.. Nat Genet.

[42] Hamilton H, Gomos J, Berns KI, Falck-Pedersen E (2004). Adeno-associated virus site-specific integration and AAVS1 disruption.. J Virol.

[43] Hüser D, Weger S, Heilbronn R (2002). Kinetics and frequency of adeno-associated virus site-specific integration into human chromosome 19 monitored by quantitative real-time PCR.. J Virol.

[44] Hüser D, Heilbronn R (2003). Adeno-associated virus integrates site-specifically into human chromosome 19 in either orientation and with equal kinetics and frequency.. J Gen Virol.

[45] Gonçalves MAFV, van der Velde I, Janssen JM, Maassen BHT, Heemskerk EH (2002). Efficient generation and amplification of high-capacity adeno-associated virus/adenovirus hybrid vectors.. J Virol.

[46] Sambrook J, Russell DW (2001). Molecular cloning: a laboratory manual, 3rd ed.

[47] Taboit-Dameron F, Malassagne B, Viglietta C, Puissant C, Leroux-Coyau M (1999). Association of the 5′ HS4 sequence of the chicken β-globin locus control region with human EF1α gene promoter induces ubiquitous and high expression of human CD55 and CD59 cDNAs in transgenic rabbits.. Transgenic Res.

[48] Gonçalves MAFV, Pau MG, de Vries AAF, Valerio D (2001). Generation of a high-capacity hybrid vector: packaging of recombinant adenoassociated virus replicative intermediates in adenovirus capsids overcomes the limited cloning capacity of adenoassociated virus vectors.. Virology.

[49] Gonçalves MAFV, van der Velde I, Knaän-Shanzer S, Valerio D, de Vries AAF (2004). Stable transduction of large DNA by high-capacity adeno-associated virus/adenovirus hybrid vectors.. Virology.

